# Personals

**Published:** 1915-04

**Authors:** 


### Personals.

Dr. C. F. Henderson of Pittsburg has been appointed county health officer by the commissioners court.

Dr. A. E. Acton has returned to his home in Comanche from a sanitarium at Temple.

Mrs. J. F. Clark, wife of Dr. Jo Clark of Iowa Park, is very ill in a sanitarium at El Paso.

Dr. J. A. L. Wolfe of Van Alstyne was injured on March 12 when his horse ran away.

Dr. A. B. Conley, former State Superintendent of Buildings and Grounds, underwent an operation on March 12 in Fort Worth, which was very successful. Dr. Conley has recently been appointed by Governor Ferguson cattle inspector for the Northern District of Texas.

Dr. P. M. Adams and wife of Freeland are receiving the congratulations of their friends over a very narrow escape from death on March 18. A horse which they were driving took fright and backed them off a bridge, plunging them about fifteen feet to the creek below. The accident occurred on South Main Street in Cleburne.

Dr. Tom Childress left Arlington on March 10 to join Mrs. Childress in Los Angeles, Cal., at which place they will be joined by their son Clyde. Dr. and Mrs. Childress plan to celebrate their forty-first wedding anniversary on March 20.

Dr. R. C. Youngblood and wife will soon take a trip to the Panama-Pacific Exposition and will return via Rochester, Minn., and Chicago, where Dr. Youngblood will do post-graduate work.

Dr. L. P. Allison of Brownwood is very ill in Dallas at the home of his brother. The doctor recently underwent an operation for appendicitis in Dallas, which was successful. A week later pleurisy developed, followed by embolic pleuro-pneumonia in both lungs. There is much anxiety over his condition.

AUBREY PHYSICIAN MARRIED IN OKLAHOMA CITY.

Dr. Henry C. Amos, a well known physician of Aubrey, was married March 11 to Miss Clytie Ingram, daughter of Mr. and Mrs. W. R. Ingram of Oklahoma City. After a short wedding journey the doctor and his bride will be at home in Aubrey.

